# Influence of image‐viewers and artifacts on implant length measurements in cone‐beam computed tomography: an *in vitro* study

**DOI:** 10.1002/cre2.18

**Published:** 2016-02-10

**Authors:** Lydia Vazquez, Murali Srinivasan, Firas Khouja, Christophe Combescure, Jean‐Pierre Carrel

**Affiliations:** ^1^ Department of Orofacial Rehabilitation, Oral and Maxillofacial Radiology University Clinics of Dental Medicine, University of Geneva Geneva Switzerland; ^2^ Division of Gerodontology and Removable Prosthodontics University Clinics of Dental Medicine, University of Geneva Geneva Switzerland; ^3^ Department of Orofacial Rehabilitation, Oral and Maxillofacial Radiology University Clinics of Dental Medicine, University of Geneva Geneva Switzerland; ^4^ CRC and Division of Clinical Epidemiology, Department of Health and Community Medicine University of Geneva and University Hospitals of Geneva Geneva Switzerland; ^5^ Division of Oral and Maxillofacial Surgery University Hospitals of Geneva Geneva Switzerland

**Keywords:** Cone beam computed tomography, dental implants, dimensional measurement accuracy, image‐viewer, radiology

## Abstract

This preclinical *in vitro* study compared the accuracy of implant lengths measured in two different image‐viewers, and examined whether implant‐induced artifacts affected the implant length measurements on CBCT images. A resin edentulous mandibular model, with multiple adjacent implants in the posterior segments, was acquired with a CBCT machine. In two different image‐viewers, two observers independently measured the implant length. Vertical measurements on CBCT images were carried out twice at each session, and repeated one week later. The results demonstrated no significant differences between actual and measured implant lengths. The differences in the mean error for vertical measurements using the two different image‐viewers (cross‐sectional images: OsiriX viewer = −0.01 ± 0.03 mm, NewTom viewer = −0.05 ± 0.09 mm, *p*‐value = 0.056; sagittal images: OsiriX viewer = −0.03 ± 0.04 mm; NewTom viewer = −0.04 ± 0.10 mm, *p*‐value = 0.24) were not statistically significant. This *in vitro* investigation suggests that the accuracy of implant length measurements on CBCT images was not influenced by image‐viewers or by the presence of implant‐induced artifacts. The presence of multiple adjacent implants in the posterior segments of the mandible is not likely to impact the measurements made between the implant apex and vital structures on CBCT images.

## Introduction

Successful implant treatment requires a systematic surgical planning entailing thorough clinical and radiographic examinations. Cone‐beam computed tomography (CBCT) is increasingly popular for implant surgery because it offers high spatial resolution, valuable data for guided surgery and virtual implant planning, and potentially lower radiation doses than computed tomography (CT) (Benavides et al. 2012). For dental implant planning in posterior segments of the mandible, the literature suggests that conventional and digital panoramic radiographs are reliable to determine the preoperative implant length when a safety margin of 2 mm above the mandibular canal is respected (Buser & von Arx 2000; Vazquez et al. 2008; Vazquez et al. 2011). Panoramic radiographs, however, cannot provide bone width evaluation, whereas 3‐dimensional acquisition of vital anatomical structures allows preoperative measurements and selection of implants of appropriate length and diameter (De Vos et al. 2009; Tyndall et al. 2012).

In accordance with ethical principles, we plan a prospective clinical trial; after insertion of two or more adjacent implants in the posterior segments of the mandible, patients will receive a postoperative CBCT examination. On the CBCT images, the implant's length, the vertical distance from the implant's tip to the mandibular canal, and the distance from the superior border of the alveolar ridge to the mandibular canal will be assessed. Measurements on CBCT images, however, can be variously impacted by metal artifacts (Schulze et al. 2010; Schulze et al. 2011) or by image‐viewing software (Gaia et al. 2013). A report on the accuracy of measurements on CBCT images concluded that measurements with Vitrea imaging software program (Vitrea 3.8.1, Vital Images Inc., Plymouth, MN) were accurate and precise, but measurements with the OsiriX viewer (version 1.4.2 64‐bit, Pixmeo, Geneva, Switzerland) were less accurate (Gaia et al. 2013). Thus, linear measurements obtained with Vitrea software (maximum difference 0.42 mm) were closer to the dry skull values than measurements (maximum difference 2.54 mm) obtained with OsiriX (Gaia et al. 2013). An ongoing study on edentulous cadaver mandibles suggested that image‐viewers can influence the accuracy of linear measurements in the posterior mandible (personal communication shared by Professor Claudio Costa, University of Sao Paulo, Brazil). If an image‐viewer has the potential to impact linear measurements on CBCT views, then not only the implant length but also the distance measured between the tip of a dental implant and the mandibular canal may be similarly impacted by the image‐viewer. In addition, implant‐induced artifacts have the capacity to affect the quality of image reconstruction and the potential to impact implant dimensions on CBCT images (Schulze et al. 2010).

In preparation for our clinical study, we conducted a preclinical *in vitro* study to compare the accuracy of implant length measurements on CBCT images using two different image‐viewers. We also examined whether implant‐induced artifacts affected the implant length measurements on CBCT images.

## Materials and Methods

### Study model

**Figure 1:**
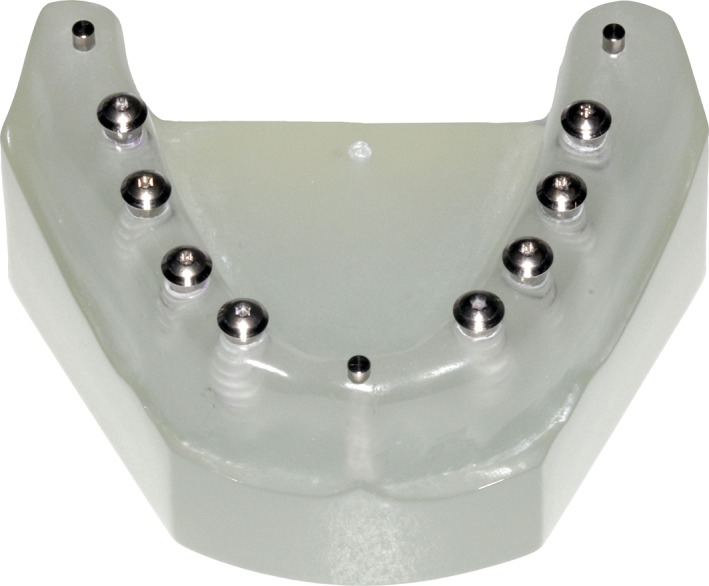
Radio‐opaque custom‐made edentulous mandibular resin model with eight tissue level implants.

A custom‐made resin model was fabricated representing an edentulous mandible (Fig. 1). Eight dental implants (Tissue Level, RN, Ø4.1 × 8 mm, and Tissue Level, RN, Ø4.1 × 10 mm, Institut Straumann AG, Basel, Switzerland) were inserted into sites corresponding to the canine, first and second premolars, and first molar. Three crestal reference pins were inserted in the model: one in the mandibular midline and two pins distal to molar implant sites. Implant covers screws were screwed in the implants.

### Cone‐beam computed tomography images

The study model was stabilized on a tripod (Manfrotto 190 XPROB, Manfrotto, Bassano, Italy) and placed in the NewTom VGi (Quantitative Radiology, Verona, Italy) CBCT machine. A radiographer specialized in CBCT imaging positioned the model in a strict horizontal position. Two CBCT acquisitions of the model were made. After the first acquisition (Acquisition 1), the model was removed from the CBCT machine and repositioned back to the original horizontal position for a second acquisition (Acquisition 2).

NNT® NewTom software (Quantitative Radiology, Verona, Italy) was used for volumetric acquisitions of the mandibular resin model. The high‐resolution mode (voxel size: 0.15 mm and field of view [FOV]: 12 × 8 cm) was used for image acquisition; exposure parameters were set at 110 kVp, 0.60 mA, 18 sec scan time and 5.4 sec X‐ray emission time. Primary reconstructions in the NNT viewer were made with an isotropic voxel with an edge length of 150 µm (pixel size 0.15 and slice thickness 0.15) using the NNT viewer. The data from the two acquisitions were saved on the NewTom VGi CBCT workstation and on a compact disk that included the NNT viewer. In a 512 × 512 matrix, the CBCT images were exported in digital imaging and communications in medicine (DICOM) format to another compact disk.

### Image‐viewers

Two image‐viewers were evaluated in this study: (1) NNT® NewTom viewer (version 3.11β5, Quantitative Radiology, Verona, Italy) on Windows Seven® (Microsoft Corporation, Redmond, USA) Dell Optiplex® 9100 computer with a 1920 × 1080 resolution LCD screen, Dell U2312HM (Dell Computer, Round Rock, USA) and (2) OsiriX DICOM viewer (version 1.2 64‐bit; Pixmeo, Geneva, Switzerland) installed in an iMac OS X (version 10.6.8; Apple Inc., Cupertino, CA) independent workstation (iMac 27‐in. Quad Core 3.4 GHz Intel Core i7) on a 2560 × 1440 resolution LCD screen.

### Measurements

The axial, sagittal, and cross sections were displayed in multi‐planar reconstruction (MPR) mode. Axial views were used to locate each implant shoulder. On the cross‐sectional and sagittal views, a horizontal line was drawn connecting the external limits of the implant shoulder (implant shoulder line). The implant length measurement was obtained by tracing a line along the long axis of each implant from the implant shoulder line to the tip of the implant. Only the posterior molar and premolar implants, used as reference objects of known dimensions, were measured for this study. The real length of implants in tooth positions 45, 34, and 36 was 11.8 mm, and the real length of implants 46, 44, and 35 was 9.8 mm. Two observers, experts in oral surgery and radiology, made the measurements twice, 1 week apart, on each image‐viewer. Each observer was calibrated to the implant height measurements on cross‐sectional and sagittal images of an implant placed in the canine region. After calibration, each observer independently made 72 individual vertical measurements of the implants positioned in the premolar and molar regions.

### Statistical analysis

The inter‐observer and intra‐observer reproducibility was assessed by Bland and Altman analysis and was plotted against the mean; the limits of agreement were reported. The limits of agreement (the interval covering approximately 95% of the differences) showed the range of differences between observers or sessions. The maximum tolerated error was set at 0.3 mm prior to the statistical analyses. For measurements with NNT viewer, the agreement between first and second acquisitions was assessed to determine whether manipulation and repositioning of the mandibular resin model impacted on the accuracy of vertical measurements.

The error of the measurement, namely the radiological implant length minus the implant's real length, was described for each software, acquisition, and view (cross‐sectional and sagittal images). Distribution of the errors was graphically represented by boxplots and described by mean, standard deviation, 95% confidence interval around the mean, median, minimal, and maximal values. Percentages of measurements with a null error were reported. A Wilcoxon test compared the absolute values of errors for OsiriX and NNT viewers. A MacNemar test compared the percentage of NNT and OsiriX measurements with a null error. Statistical analyses were repeated after rounding the OsiriX measurements to the nearest tenth for comparison with NNT viewer, which reports values only to a single digit after the decimal. This verified that the better accuracy obtained with OsiriX was caused not by the number of digits but by the measurement software. A bio‐statistician (C. C.) did the statistical analyses in S‐PLUS software (version 8.0, Insightful Corp, Seattle, WA).

## Results

### NNT viewer

**Figure 2:**
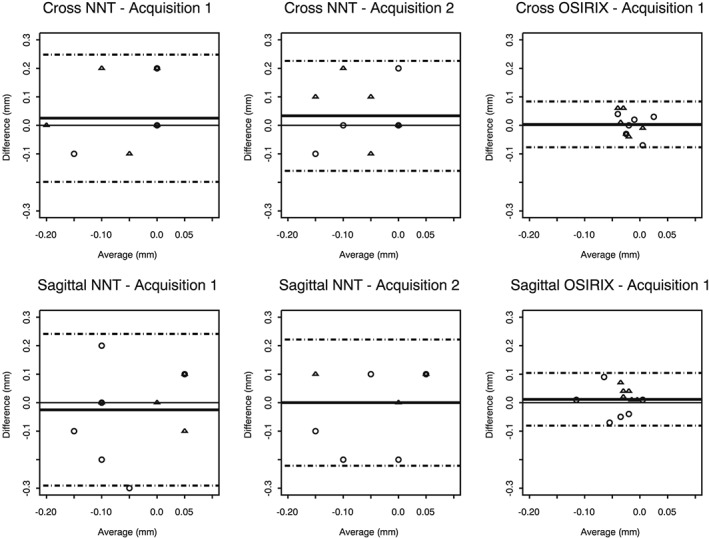
Bland–Altman analysis showing the intra‐observer agreements for the measurement errors.

**Figure 3:**
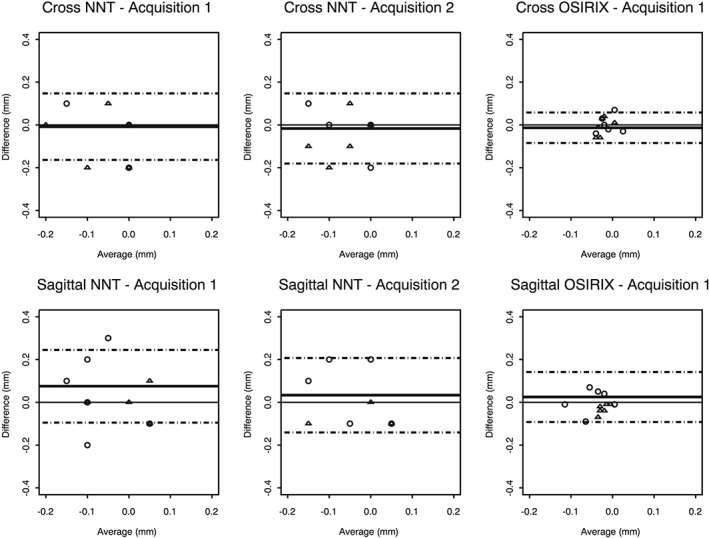
Bland–Altman analysis showing the inter‐observer agreements for the measurement errors.

The error calculated for all measurements, defined as the difference between the measured radiological value and the real implant length, is described in Table 1. Individual errors did not exceed −0.20 mm and +0.10 mm. On average, the implant length was underestimated by 0.05 mm on cross‐sectional images and by 0.04 mm on sagittal images. Measurements between sessions (Fig. 2) and between observers (Fig. 3) were similar. The mean difference was close to zero, and the limits of agreement did not exceed ± 0.30 mm. The second acquisition (after repositioning) produced similar results (Table 1 and Figs. 2, 3). Furthermore, the differences between measurements for first and second acquisitions were less than 0.01 mm on average for cross‐sectional and sagittal views, and no differences exceeded ± 0.10 mm. These results found that manipulation of the model had no sensitive impact on the accuracy of vertical measurements. Inter‐observer and intra‐observer reproducibility was acceptable with the NNT viewer.

**Table 1:** Descriptions of error in the measurements of implant lengths.

Acquisition	Software	View	Mean error in mm (standard deviation)	95% CI around the mean	Median error in mm [minimal–maximal]	% of measures with a null error
1	NNT	Cross	−0.05 (0.09)	[−0.09;−0.02]	0.00 [−0.20;0.10]	12/24 (50)
1	NNT	Sagittal	−0.04 (0.10)	[−0.08;0.00]	0.00 [−0.20;0.10]	11/24 (45.8)
2	NNT	Cross	−0.05 (0.08)	[−0.08;−0.02]	0.00 [−0.20;0.10]	13/24 (54.2)
2	NNT	Sagittal	−0.03 (0.09)	[−0.07;0.00]	0.00 [−0.20;0.10]	13/24 (54.2)
1	OSIRIX	Cross	−0.02 (0.03)	[−0.03;−0.01]	−0.02 [−0.07;0.04]	4/24 (16.7)
1	OSIRIX	Sagittal	−0.04 (0.04)	[−0.05;−0.02]	−0.02 [−0.12;0.01]	5/24 (20.8)
1	OSIRIX *	Cross	−0.01 (0.03)	[−0.03;0.00]	0.00 [−0.10;0.00]	21/24 (87.5)
1	OSIRIX *	Sagittal	−0.03 (0.04)	[−0.04;−0.01]	0.00 [−0.10;0.00]	18/24 (75.0)

* Rounded off to the first decimal.

### OsiriX viewer

Similarly to NNT measurements, measurement errors with OsiriX viewer were close to zero on average. But errors clustered more narrowly around zero with OsiriX than with NNT viewer (Table 1): measurement errors ranged from −0.07 to 0.04 mm on cross sections and from −0.12 to 0.01 mm on sagittal views. On cross sections, the absolute values of errors with OsiriX viewer were significantly lower than the absolute values of errors with NNT viewer (*p* = 0.04). The trend was similar for sagittal images but did not reach statistical significance (*p* = 0.13). These results indicated that the measurements of the implant length were more accurate with OsiriX viewer than with NNT viewer. A multivariate regression model found that the difference of errors between OsiriX and NNT viewer was not associated with the observer, the session, the jaw side, or the implant site (Table 2). For the differences between sessions (Fig. 2) and between observers (Fig. 3), Bland and Altman analysis showed narrower limits of agreement with OsiriX viewer. Our initial decision to measure the implant lengths on one, not both acquisitions with the OsiriX viewer, was validated by the differences found between measurements for the first and second acquisitions not exceeding ± 0.10 mm with the NNT viewer.

**Table 2:** Multivariate regression to explore associations between factors, and differences in measurement errors between the two image‐viewers.

Factors	Difference between NNT and Osirix			
	Cross		Sagittal	
	Estimates (standard error)	*p*‐value	Estimates (standard error)	*p*‐value
Intercept	−0.05 (0.05)	0.26	−0.04 (0.05)	0.43
Observer				
First	0 (reference)		0 (reference)	
Second	0.01 (0.04)	0.90	0.05 (0.04)	0.21
Session				
First	0 (reference)		0 (reference)	
Second	0.02 (0.04)	0.58	−0.04 (0.04)	0.36
Location				
Molar	0 (reference)		0 (reference)	
Premolar	0.04 (0.04)	0.33	0.06 (0.04)	0.19
Side				
Right	0 (reference)		0 (reference)	
Left	0.05 (0.04)	0.23	−0.02 (0.04)	0.67

## Discussion

Cone‐beam computed tomography systems have several types of X‐ray detectors and an array of FOV, voxel sizes, imaging protocols, acquisition times, and exposure parameters. In addition, various image reconstruction algorithms and different measurement software programs are used. As a result, different image‐viewing systems measuring CBCT images have reported a range of accuracies (Baumgaertel et al. 2009; Maloney et al. 2011; Gaia et al. 2013; Kosalagood et al. 2015). This preclinical CBCT study investigated the impact of two image‐viewers on vertical linear measurements, namely the implant length, and whether the distance measured between a dental implant's tip and the mandibular canal might similarly be impacted. The CBCT manufacturer's dedicated NNT viewer and the OsiriX DICOM viewer were used for vertical measurements. Measured values were compared with the reference value for each implant length. Implant length values measured with OsiriX viewer, rounded to the first decimal for comparison with the values measured with the NNT viewer, were identical for rounded and non‐rounded values. Measurements were slightly more accurate with the OsiriX viewer than with the NNT viewer. OsiriX viewer (rounded values) yielded mean measurement differences of −0.01 ± 0.03 mm (range −0.10 to 0.00 mm) in cross‐sectional images and 0.03 ± 0.04 mm (range −0.10 to 0.00 mm) in sagittal images. NNT viewer yielded mean measurement differences of −0.05 ± 0.09 mm (range −0.20 to 0.10 mm) in cross‐sectional views and 0.03 ± 0.09 mm (range −0.20 to 0.10 mm) in sagittal views. These measurement differences, obtained with two viewers on images acquired with an NNT VGi CBCT (FOV of 120 × 80 mm and voxel size of 0.15 mm) are similar to the values reported by a high‐resolution large FOV CBCT study (Kosalagood et al. 2015). Our results are also corroborated by a CT study showing a very high reliability using OsiriX viewer (Kim et al. 2012). In contrast, a CBCT study using two DICOM viewers concluded that linear measurements obtained with Vitrea software were accurate and precise, but measurements obtained with the OsiriX viewer were less accurate (Gaia et al. 2013). Moreover, a viewer by another manufacturer (CB Mercuray, Hitachi Medical Systems, Tokyo, Japan) generated significantly underestimated values (absolute measurement errors ranging from ‐2.56 to ‐0.63 mm, mean 1.74 ± 0.73 mm) (Kosalagood et al. 2015). In our model study, the OsiriX viewer in MPR mode had a slight practical advantage, which was to allow the observers to measure the implants directly in the cross‐sectional and sagittal windows, unlike the NNT viewer that required cropping of cross‐sectional and sagittal images before implant length measurements.

Another aim of this study was to investigate the potential impact of implant‐induced artifacts on the implant length. Authors reporting on CBCT dimensional accuracy have used cylindrical titanium markers (Lagravere et al. 2010) and orthodontic wires as radiopaque markers (Ludlow et al. 2007). The risk for streak artifacts and beam‐hardening artifacts increases in the presence of metal and titanium implants (Schulze et al. 2010; Schulze et al. 2011; Kamburoglu et al. 2014). The titanium Straumann dental implants and the three crestal titanium pins in our investigation resulted in visible metal artifacts; dental implants did not appear circular on axial images and dark streaks appeared between the implants on sagittal sections. When measuring, a small difficulty in identifying the exact endpoint of the implants was encountered but did not appear to influence measurement accuracy. The results of this *in vitro* study suggest that the metal artifacts did not impact the accuracy of the measured implant lengths. Hence, in the clinical trial examining patients with multiple posterior mandibular implants, the presence of implants is not expected to impact the distance measured between the tip of the implant and the mandibular canal on CBCT images. Nonetheless, these findings require careful consideration in clinical applications because teeth, bone, soft tissues, and restorative materials that affect image quality were not simulated in this *in vitro* experiment.

The methodology of this preclinical study is sound, but experimental limitations remain. This study was limited to the assessment of vertical measurements on two different implant lengths (8 mm and 10 mm), which are the lengths favored by many surgeons for posterior mandibular implant sites (Vazquez et al. 2008). As only two implant lengths were assessed, measurement inaccuracies may have been underpowered. A second limitation might be the static model used for measurements instead of a patient. The high‐resolution acquisitions in this study, requiring longer scan times and an increased number of images, add susceptibility to patient movement during acquisition (Schulze et al. 2011). Our results showed good measurement accuracy on a static model. A change in patient positioning for the CBCT acquisition can alter the vertical measurements on CBCT images (Tomasi et al. 2011; Visconti et al. 2013). To mimic change in head positioning, we removed the model and repositioned it before a second acquisition. Repositioning the model did not influence the accuracy of vertical measurements under the experimental conditions of this *in vitro* study. Another possible limitation could have been the influence of voxel size, exposure parameters, and FOV, which were not analyzed. Present results apply only to the specific machine and settings under investigation. We studied one FOV size and acquisition mode in preparation for a clinical study. Future studies might evaluate other acquisition modes available with the NewTom VGI.

A further limitation of this study may have been due to the impact of measurement capabilities of the image‐viewers. We enquired with the respective manufacturers about possible discrepancies in the software viewers' measurement capabilities and other interferences from the viewing software. Unfortunately, we did not receive an answer on these technical questions. To the best of our knowledge, compensation features for measurement capabilities discrepancies of software programs do not appear to exist. In this study, the measurement differences observed between the examiners and between the image‐viewers were statistically insignificant, which suggests that the incremental measurement error (if any) was unlikely to have an impact. The assessment of the incremental measurement error is beyond the scope of our preclinical pilot study.

In conclusion, this *in vitro* investigation suggests that the accuracy of implant length measurements on CBCT images was not influenced by image‐viewers or by the presence of implant‐induced artifacts. Therefore, the presence of multiple adjacent implants in the posterior segments of the mandible is not likely to impact the measurements made between the implant apex and vital structures on CBCT images. These results should be further evaluated in clinical studies.

## Conflict of interest

The authors declare that they have no conflict of interest.

This article does not contain any studies on human or animal subjects performed by any of the authors.
